# Role of Corticosteroids in Drug-Induced Liver Injury. A Systematic Review

**DOI:** 10.3389/fphar.2022.820724

**Published:** 2022-02-10

**Authors:** Einar S. Björnsson, Vesna Vucic, Guido Stirnimann, Mercedes Robles-Díaz

**Affiliations:** ^1^ Faculty of Medicine, University of Iceland, Reykjavik, Iceland; ^2^ Department of Gastroenterology, Landspitali University Hospital Reykjavik, Reykjavik, Iceland; ^3^ Department of Nutritional Biochemistry and Dietology, Centre of Research Excellence in Nutrition and Metabolism, National Institute for Medical Research, University of Belgrade, Belgrade, Serbia; ^4^ Hepatology, Departement for Visceral Surgery and Medicine, University Hospital Inselspital and University of Bern, Bern, Switzerland; ^5^ Unidad de Gestión Clínica de Aparato Digestivo, Instituto de Investigación Biomédica de Málaga-IBIMA, Hospital Universitario Virgen de la Victoria, Facultad de Medicina, Universidad de Málaga, Centro de Investigación Biomédica en Red de Enfermedades Hepáticas y Digestivas (CIBERehd), Málaga, Spain

**Keywords:** DILI, corticosteroids, treatment of DILI, acute liver failure, drug-induced autoimmune hepatitis, drug-induced liver injury, check point inhibitors

## Abstract

**Introduction:** Apart from cessation of the implicated agent leading to drug-induced liver injury (DILI), there is no standard therapy for DILI. Corticosteroids have been used in DILI, although their efficacy is unclear. Published data showed either beneficial effects or no improvement associated with steroid therapy. The aim of the current study was to perform a systematic review of the role of corticosteroids in the treatment of DILI.

**Methods:** A search was performed in PubMed, searching for the terms: “corticosteroids” and “drug-induced liver injury”. Observation studies were included, but case reports excluded.

**Results:** A total of 24 papers were retrieved. Most of these were observational studies on the effects of corticosteroids in moderate/severe DILI (n = 8), reports on the corticosteroid treatment in patients with drug-induced autoimmune hepatitis (DI-AIH) (n = 5), and effects of corticosteroids in drug-induced fulminant acute liver failure (ALF, n = 2). Furthermore, treatment of corticosteroids in patients with liver injury due to check point inhibitors (CPIs) was addressed in nine studies. In moderate/severe DILI, six out of eight studies suggested steroid treatment to be beneficial, whereas two studies showed negative results. All five observational studies on the effects of corticosteroids in DI-AIH showed good therapeutic response with rapid and long lasting effects after discontinuation of corticosteroids and without evidence of relapse. Steroid therapy was not associated with improved overall survival in patients with drug-induced fulminant ALF. CPIs-induced liver injury was found to improve spontaneously in 33–50% without corticosteroids, and the rate of patients who were treated responded to steroids in 33–100% (mean 72%).

**Conclusions:** The majority of studies analyzing the effects of corticosteroids in moderate/severe DILI have demonstrated beneficial effects. However, this was not the case in drug-induced fulminant ALF. Patients with DI-AIH had an excellent response to corticosteroids. The majority of those with CPIs-induced liver injury responded to corticosteroids; however, patients without treatment usually recovered spontaneously. The observational design and comparison with historical controls in these studies makes it very difficult to draw conclusions on the efficacy of corticosteroids in DILI. Therefore, there is a strong need for a randomized controlled trial to properly assess the role of corticosteroids in DILI.

## Introduction

Corticosteroids or steroids have been used for several decades in the treatment of autoimmune hepatitis (AIH). Their use is based on placebo-controlled trials dating back to the 1970s. In an early clinical trial including patients with chronic active HBsAg-negative hepatitis, treatment consisted of prednisolone or placebo. In this early trial, 5-years survival was 32% in patients on placebo but 82% in steroid treated patients (Kirk et al., 1980). Many patients with suspected drug-induced liver injury (DILI) have histological features that are similar to those observed in AIH (Suzuki et al., 2011; Germani et al., 2021). It is therefore conceivable that patient with DILI might benefit from steroid treatment as well, but data on corticosteroid treatment in DILI is scarce. Apart from AIH, alcoholic hepatitis and acute liver graft rejection in transplanted patients, there are no well documented indications for steroid therapy in patients with liver disease.

## Material and Methods

A systematic review of the published literature was performed with a search of https://pubmed.ncbi.nlm.nih.gov/(via MEDLINE up to October 2021). This systematic review was performed following the PRISMA guidelines. The search was conducted with the following main terms: “Corticosteroids”, combined with “drug-induced liver injury”. All types of design of clinical human studies with the administration of corticosteroids in DILI were analyzed, provided that the etiology of liver disease was either conventional drug-induced or related to herbal and dietary supplements (HDS).

Included were articles in English assessing the effects of corticosteroids in the treatment of patients diagnosed with DILI due to conventional drugs and HDS; Exclusion criteria were review articles, animal studies and/or basic research studies and liver injury due to other causes than DILI. Furthermore, case reports describing effects of corticosteroids in DILI were excluded.

All publications retrieved in the search were screened for eligibility by different authors (ESB, VV, GS and MR) based on the predetermined criteria listed above. Discrepancies were resolved by majority opinion. The work was supervised by a senior investigator (ESB).

The following data were extracted from the included publications: surname of the first author, year of publication, number of patients, design of the study, type and severity of DILI, doses of corticosteroids given, the duration of therapy and treatment response in terms of numerical values. In patients with acute fulminant liver failure, survival was considered as the treatment response in contrast with the other studies, where normalization of liver tests were considered treatment response, lack of relapse of hepatitis after stopping corticosteroids and no need for additional immunosuppressive drugs.

## Results

**FIGURE 1:**
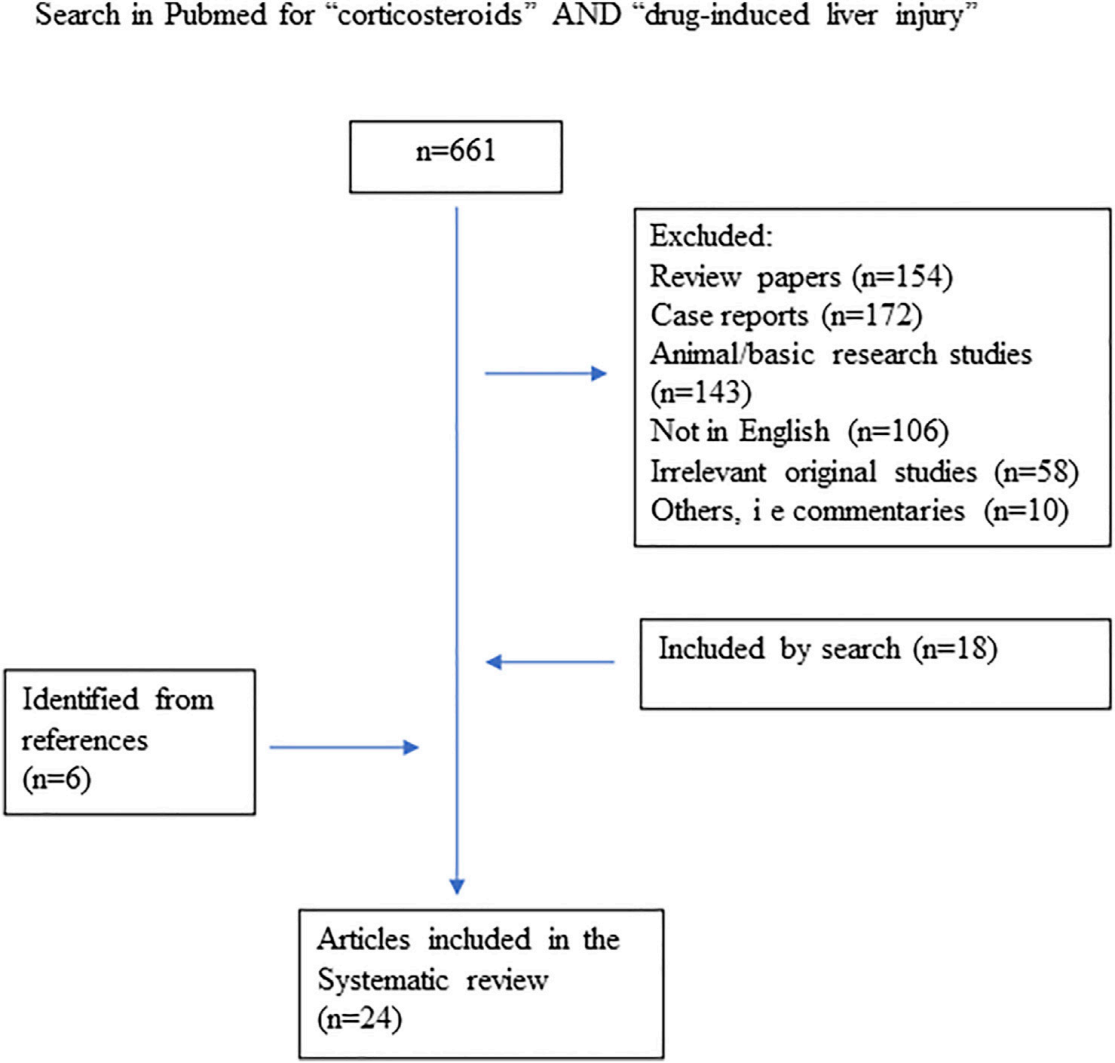
Flow chart over the literature review process.

A total of 661 publications were retrieved from the database search. Of them, 643 publications were not eligible for the following reasons: being review papers, case reports, animal studies, in other languages than English or being otherwise irrelevant for the aim of the study (Figure 1). After reviewing the references of the included studies and reviews identified in the literature search, six additional publications were retrieved.

Thus, the search revealed a total of 24 studies that fulfilled the predetermined criteria and were analyzed. Most of these were observational studies and clinical studies on the effects of corticosteroids in moderate and severe DILI (n = 8), reports on the corticosteroid treatment in patients with drug-induced autoimmune hepatitis (DI-AIH) (n = 5), and effects of corticosteroids in drug-induced fulminant acute liver failure (n = 2). Furthermore, treatment of corticosteroids in patients with liver injury due to check point inhibitors was addressed in nine studies. In moderate/severe DILI, six out of eight studies suggested steroid treatment to be beneficial, whereas two studies showed negative results. All five observational studies in DI-AIH showed good therapeutic effects of corticosteroids. Steroid therapy was not associated with improved overall survival or improved survival in patients with drug-induced fulminant acute liver failure. Patients with check point inhibitors-induced liver injury were found to improve spontaneously in 33–50% without corticosteroids, but most of those who were treated responded to steroid therapy.

### Steroid Use in Patients With Moderate and Severe DILI

Ferrero et al. treated five patients with imatinib induced liver injury with corticosteroids with an excellent response but unfortunately without a control group (2006).

Controlled clinical studies investigating corticosteroid treatment in severe DILI are largely lacking and no placebo-controlled study has been undertaken to date. In a report by Wree et al. (2011) on 15 patients with DILI, nine patients were treated with steroid step-down therapy for several weeks, whereas six patients were treated with a steroid pulse therapy for 3 days (Table 1). Ursodeoxycholic acid (UDCA) was used in all patients. Steroids were given in high doses (2–20 mg/kg/day) and patients who received steroids had a decrease in bilirubin and transaminases to less than 50% of peak value in 2 weeks with normalization all within 8 weeks (Wree et al., 2011). The authors compared the decrease of peak bilirubin level by 50% after detection of DILI with a report from the DILIN cohort among 220 patients with DILI (Chalasani et al., 2008). In the study by Wree et al. (2011) the median duration from peak bilirubin value to 50% reduction was 8.5 days, which was significantly (*p* < 0.05) shorter than in the DILIN cohort (Chalasani et al., 2008) which had a median of 13 days. Patients without histological signs of pre-existing liver damage were found to have the most favourable clinical course (Wree et al., 2011). Interestingly, five of these patients developed DILI due to use of anabolic steroids, two as a consequence of phenprocuomon treatment, and one each because of treatment with desflurane, ovarian stimulation for *in vitro* fertilisation, flutamide, occupational solvent inhalation, trimethoprim-sulfamethoxazole, propofol, minocycline and nitrofurantoin (Wree et al., 2011). The last two mentioned drugs, nitrofurantoin and minocycline, have been associated with drug-induced autoimmune hepatitis that often require corticosteroids for improvement. As the control group consisted of historical controls with other drugs leading to DILI, the efficacy of steroids in severe DILI is not clear.

**TABLE 1:** Studies evaluating corticosteroids in different types of DILI are illustrated. The design of the study, the proportion treated with corticosteroids, the control group, doses and median duration of corticosteroids and the overall effects are shown.

	Type of DILI	Design	Number of patients treated with steroids	Control group	Dose and duration of therapy	Respone
Ferrero et al. (2006)	Imatinib ind; DILI	Observational	5/5 (100%)	None	20–40 mg, 20–24 weeks	100%
Wree et al. (2011)	Mod./severe	Observational, All were on UDCA	15/15 (100%)	Historical control	2–5 mg/kg or 15–20 mg/kg # Several weeks	100%
	DILI					
Hou et al. (2012)	Mod./severe	Observational	33/300 (11%)	Non-steroid therapy	-	100%
	DILI	Patients had jaundice			-	
Hu et al. (2016)	Mod./severe	Observational	53/203 (26%)	Non-steroid therapy	40–60 mg or 60–120 mg, {Several weeks	90.6%
	DILI	Patients had jaundice				
Pang et al. (2018)	Severe DILI	Observational	32/32 (100%)	Non-steroid therapy	-	94%
		Severity score 3				
Borlak et al. (2018)	Flupirtine	Observational	21/21 (100%)	Historical controls	1 mg/kg	90.5%
		All were on NAC			“stopped when ALT normalized”	
Wan et al. (2019)	Mod./severe	Observational	66/90 (73%)	Non-steroid therapy	40 mg	100%
	DILI				13 weeks	
Weber et al. (2019)	Mod./severe	Observational	21/21 (100%)	Patients with AIH	60 mg	91%
	DILI				19 weeks	
Björnsson et al. (2010)	DI-AIH	Observational	24/24 (100%)	Patients with AIH	20–40 mg, 8 weeks	100%
Ghabril et al. (2013)	DI-AIH	Observational	12/34 (35%)	No controls	-	100%
Rodrigues et al. (2015)	DI-AIH	Observational	8/8 (100%)	No controls	-	100%
Björnsson et al., 2017	DI-AIH	Observational	9/15 (60%)	No controls	20–40 mg, 8 weeks	100%
Björnsson et al. (2022)	Infliximab ind. DILI	Observational	17/36 (47%)	Non-steroid therapy	20–40 mg, 8 weeks	100%
Rakela et al. (1991)	ALF due to drugs	Observational	44/62 (71%)	Placebo	400–800 mg*	9–24%
					-	
Karkhanis et al. (2014)	ALF due to drugs	Observational	25/66 (38%)	Non-steroid therapy	42 mg	61%
					5 weeks	
Huffman et al. (2018)	DIILI due to CPIs	Observational	16/17 (94%)	No controls	1 mg/kg/day	81%
					6 weeks	
De Martin et al. (2018)	DILI due to CPIs	Observational	10/16 (63%)	Non-steroid therapy (37%)	1 mg/kg/day	90%
					-	
Cheung et al., 2019	DILI due to CPIs	Observational	19/21 (90.5%)	No controls	1 mg/kg/day	53%
					50–60 mg	
Gauci et al. (2018)	DILI due to CPIs	Observational	5/10 (50%)	Non-steroid therapy	1 mg/kg/day	100%
					7 weeks	
Riveiro-Barciela et al. (2020)	DILI due to CPIs	Observational	28/28 (100%)	Patients with AIH	60 mg	64%
					9 weeks	
Miller et al. (2020)	DILI due to CPIs	Observational	67/100 (67%)	Non-steroid therapy (33%)	-	86%
					6 weeks	
Kitagataya et al. (2020)	DILI due to CPIs	Observational	4/17 (24%)	No controls	1–2 mg/kg/day	50%
					-	
Ito et al. (2021)	DILI due to CPIs	Observational	30/58 (52%)	No controls	0.5–2.0 mg/kg/day	33%
Gauci et al. (2021)	DILI due to CPIs	Observational	13/21 (62%)	Non-steroid therapy (38%)	1 mg/kg/day	92%
					7 weeks	

DILI, drug-induced liver injury.

UDCA, urso deeoxycholic acid, NAC = N-acetylcystein, ALF, acute liver failure; CPIs, Check Point Inhibitors.

#9 were treated with a steroid step-down therapy with reduction of the daily dose over several weeks (prednisone, range 2–5 mg/kg/day and weekly reduction); six patients received a steroid pulse-therapy for 3 days (prednisone, range 15–20 mg/kg/day).

{ Two methods of drug administration. (1) Corticosteroid step-down therapy with a reduction of the daily dose over several weeks (methylprednisolone 60–120 mg/day or prednisone 40–60 mg/day for 3–5 days, and then prednisone at 20 mg/day, and tapered for 5–10 mg weekly thereafter). (2) Corticosteroid pulse therapy, using methylprednisolone 60–120 mg/day for 3–5 days.

Researchers from Beijing reported beneficial effects of steroids in patients with DILI, too. Among 70 patients with high bilirubin (TBIL ≥10× ULN), 20 were treated with steroid step-down therapy (79 ± 26 days) and others with non-steroid therapy (Hou et al., 2012). The steroid therapy group showed a higher DILI resolution rate (*p* = 0.029) and a shorter recovery time (*p* = 0.012) (Hou et al., 2012) (Table 1).

In a study from Shanghai with 203 DILI patients of whom 53 received steroids but other than that the same management than the non-steroid group (Hu et al., 2016), corticosteroid therapy was associated with improvement in recovery rate from 77 to 88% in the higher 50% quartile of total bilirubin (TB) values (Table 1). Administration of corticosteroids was found to hasten the resolution of liver injury by shortening the reduction of TB to 50% of peak value from 17 to 12 days (*p* < 0.05). The interpretation by the authors was that corticosteroids were beneficial in patients with severe DILI (TB > 243 μmol/L) who might be at risk of developing ALF (Hu et al., 2016).

However, Pang et al. (2018) reported the results of thirty-two acute DILI patients who received steroid therapy. In these patients no improvement in the recovery time of ALT or resolution rate was observed compared with the non-steroid group (with otherwise no difference in their management). Thus, the authors concluded that steroid therapy was not associated with an improved recovery time or survival in acute severe DILI patients (Pang et al., 2018) (Table 1).

Borlak et al. from Germany (2018) recently analyzed in a retrospective cohort of patients with flupirtine induced liver injury the effects of oral prednisolone in addition to N-acetyl-cystein (NAC) (Table 1). Overall, 21 patients with liver injury associated with flupirtine received NAC/prednisolone and outcome in these patients was compared with an external cohort of 30 cases of flupirtine induced liver injury not receiving this treatment or another therapy (Borlak et al., 2018). The combined treatment was well tolerated and was associated with significant ALT, AST and INR improvement within 2 weeks, but patients with jaundice resolved slowly. However, two patients who were said to have started medical treatment late in the course of the liver injury, developed hepatic encephalopathy and required liver transplantation. Normalization in liver tests occurred more rapidly compared with the historical controls including a case with fatal outcome (Borlak et al., 2018). Additional *in vitro* studies revealed glutathione depletion as an important contribution to the development of DILI (Borlak et al., 2018). As the authors pointed out, prospective randomized clinical trials are needed to confirm efficacy of this treatment in severe DILI.

However, a recent study from China could not reproduce these results (Wan et al., 2019) (Table 1). Overall, 66 out of 90 DILI patients enrolled in this study were treated with steroids, while 24 composed the control group without other medical therapy. Prednisone therapy was not beneficial at a median daily dose of 40 mg for the treatment of severe DILI. When the prednisone group was divided into two subgroups, the high dose group (>40 mg per day) was associated with lower rates of severity reduction than the control group or the low dose group (<40 mg per day) (Wan et al., 2019).

In a recent study from Germany, patients suspected of DILI with acute liver injury (ALI) who were referred to a single centre were assessed in terms of ALT response to corticosteroids (Weber et al., 2019) (Table 1). Among 44 patients with ALI who took at least one drug and who were treated with steroids, 22 had the final diagnosis of AIH and 22 were considered to have DILI. Scores of AIH and for DILI were calculated at baseline. The decrease in ALT levels was significantly more marked in the patients with the final diagnosis of DILI than in the AIH group. This is somewhat surprising as AIH patients usually respond very well to steroids. However, among the causative agents, minocycline and pembrolizumab are well documented to induce drug induced AIH (DI-AIH). It is also conceivable that there was some selection among those with a suspicion of DILI who received steroids i.e., 44/288 (15%) of all patients referred to the study on *idiosyncratic* DILI. For example, 95% of the DILI patients treated with steroids had hepatocellular type of injury (Weber et al., 2019).

Thus, is it difficult to draw firm conclusions on the beneficial effects of steroids in DILI in general, but the results are intriguing.

### Steroids in Drug-Induced Autoimmune Hepatitis

Steroids have generally been reported to be beneficial in patients with drug-induced autoimmune hepatitis (Björnsson et al., 2010; Ghabril et al., 2013; Björnsson et al., 2015; Rodrigues et al., 2015; Björnsson et al., 2017; Björnsson et al., 2022) (Table 1). In a study from the DILIN study group in the US, six patients with TNF-alpha inhibitors induced liver injury and additional 28 patients from the literature were analyzed: infliximab (n = 26), etanercept (n = 4) and adalimumab (n = 4) (Ghabril et al., 2013). A total of 22 patients had autoimmune features and 12 patients (55%) received corticosteroid therapy and responded well to therapy. In a study from Portugal with eight patients with TNF-alpha inhibitors induced liver injury (infliximab = 7, adalimumab = 1), all were treated with corticosteroids and had normalization of liver tests and those who discontinued immunosuppression did not have a relapse of AIH (Rodrigues et al., 2015).

At the current time there is no consensus on the definition of DI-AIH and most studies have presented patients with DI-AIH with a clinical diagnosis of DI-AIH with positive autoantibodies and/or IgG (Björnsson et al., 2010; Ghabril et al., 2013; Rodrigues et al., 2015; Björnsson et al., 2022). However, in one study DI-AIH was defined as a drug reaction with well-documented cause of DI-AIH, with positive ANA or SMA or elevated IgG, 10 times upper limit of normal ALT, with hepatocellular (HC) or mixed pattern or requirement of corticosteroids in patients with hepatocellular type, defined as no improvement in ALT and AST after discontinuation of the implicated agent (Björnsson et al., 2017).

Among patients from a single center study of 36 patients with infliximab induced liver injury, 17 (47%) were treated with corticosteroids (Björnsson et al., 2022). Treatment response was good with prompt resolution of liver test abnormalities and liver enzymes normalized in all patients, which was faster than in those who did not receive steroids (Björnsson et al., 2022). The relapse of liver injury was not observed after tapering of corticosteroids despite prolonged follow-up and no patients developed DILI due to a second biologic.

Patients with DI-AIH induced by drugs with well documented ability to induce AIH such as minocycline, hydralazine, methyl-dopa, nitrofurantoin and infliximab, mimic regular AIH clinically, biochemically and immunologically. Thus, as autoimmune features cannot distinguish reliably between DILI and genuine AIH in these patients, steroid treatment is recommended according to clinical guidelines (European Association for the Study of the Liver, 2015).

### Steroid Use in Acute Liver Failure

Drugs are a common cause of acute liver failure (ALF) (Ostapowicz et al., 2002; Björnsson et al., 2005; Wei et al., 2007; Reuben et al., 2010). Some of the patients with ALF have prominent inflammatory features and more than 3 decades ago steroids were initially investigated in ALF (Ware et al., 1974; European Association for the Study of the Liver, 1979; Rakela et al., 1991). These trials failed to show a benefit of steroid treatment compared with placebo. In a randomized, un-blinded clinical trial carried out in 17 European centres, ALF developed mostly due to viral hepatitis or presumed viral hepatitis. Patients were given 400 mg hydrocortisone twice a day or no medical therapy (placebo was not used). Only two patients had drug-induced ALF, both in the control group (European Association for the Study of the Liver, 1979). No significant difference in survival between the two groups was achieved. Another steroid trial in ALF from the US included 62 patients with fulminant drug-induced hepatotoxicity (Rakela et al., 1991) (Table 1). Patients received placebo, 400 mg hydrocortisone, or 800 mg hydrocortisone, and had a survival rate of 22%, 9% and 24%, respectively. Based on this study, the European Association for the Study of the Liver recommended discontinuing steroid treatment in patients with ALF.

Auto-antibodies are frequent in patients with ALF. Bernal et al. found autoantibodies in 32% of ALF patients (Bernal et al., 2007). While autoantibodies were absent in patients with ALF induced by paracetamol, they could be detected in 43% of non-paracetamol ALF patients. AIH scores classified 50% of cryptogenic cases as ‘probable autoimmune hepatitis’ (Bernal et al., 2007).

Analysis undertaken by the investigators of the Acute Liver Failure Study Group (ALFSG) suggested that many patients with ALF of indeterminate etiology had histological features indicating an autoimmune pathogenesis (Stravitz et al., 2011). This motivated members of the ALFSG to conduct a retrospective analysis of ALF patients from 1998–2007 (Karkhanis et al., 2014) (Table 1). Overall, 361 patients with ALF were divided into three subgroups based on origin of liver disease: autoimmune, DILI and indeterminate ALF. In the group consisting of 131 patients with ALF due to DILI, only 16 were treated with steroids and 115 received no steroids. Among those classified as autoimmune ALF, 25/66 (38%) were treated with steroids and of those with ALF of indeterminate etiology 21/164 (13%) (Karkhanis et al., 2014). Steroid therapy was not associated with improved overall survival or improved survival in any of these categories. In those patients with highest Model for End stage Liver disease (MELD) scores, steroid treatment was even associated with a worse outcome. Steroid therapy was associated with a marginal benefit in spontaneous overall survival (35% vs. 23%, *p* = 0.047), but this benefit did not persist in a multivariate analysis (Karkhanis et al., 2014). As the authors pointed out, it is conceivable that the lack of benefit of steroid use might be due to selection bias. Thus, patients who received steroids might have been more ill. However, MELD scores were similar in the two groups and the international normalized ratio of prothrombin time (INR) was actually higher in those who did not receive steroids. Only patients with alanine aminotransferase (ALT) in the higher 50% of values who received steroids had improved spontaneous survival (54% in patients with steroid treatment vs 25% in patients who did not receive steroids, *p* = 0.003). The authors postulated that higher ALT might be a surrogate marker for an inflammatory process that might be sensitive to corticosteroid therapy (Karkhanis et al., 2014).

### Steroid Use in Check Point Inhibitor Induced Liver Injury

In a recent review, a third type of DILI was introduced, named the indirect type (Hoofnagle and Bjornsson, 2019). This type of DILI is different from direct DILI (such as paracetamol-induced liver toxicity) and *idiosyncratic* DILI as it has to do with the action of the drug i. e. what the drug does and not what it is. In other words, the action of the drug can affect the balance in the immune system and therefore induce various immune mediated adverse effects. Most commonly implicated agents in indirect DILI are antineoplastic agents such as Check Point Inhibitors (CPI) and other monoclonal antibodies such as infliximab (Björnsson et al., 2022). In a study of 17 melanoma patients with DILI due to CPIs, other concomitant immune-mediated adverse effects were observed in 47% of patients (gastrointestinal, dermatological, endocrine, lung disorders) (Huffman et al., 2018). Similar results have been reported from larger cohorts (Miller et al., 2020). The frequency of ALT elevations in clinical trials has been found to be 3–15%, some of them of transient nature, and an increase of 5-20xULN has been observed in up to 3% of patients (Shah et al., 2020).

In the study of Huffman et al. (2018), 17 patients had any kind of hepatotoxicity (mild, moderate or severe DILI, Table 1). Twelve of 17 were diagnosed after treatment with ipilimumab, three were diagnosed after pembrolizumab, and 2 after ipilimumab combined with nivolumab. Patients were most commonly treated with systemic corticosteroids such as prednisone. Immunosuppression was tapered over a median of 42 days; in three patients steroids had to be reinitiated due to clinical or laboratory worsening of liver enzymes. Normalization of liver tests was seen within a median of 1 month after start of immunosuppression.

In retrospective studies, grade 3–4 ALT elevation were observed in 2–8% of patients (De Martin et al., 2018; Parlati et al., 2018; Miller et al., 2020). Time to onset varied from 2–24 weeks after initiation of treatment (median 4–16 weeks). Monotherapy with ipilimumab was associated with a higher incidence DILI than other CPIs, but combination of more than one CPI seems to increase the risk of DILI (Shah et al., 2020). DILI due to CPIs has a distinct biochemical and histological phenotype, and often occurs together with other immune-mediated adverse reactions. Although considered to be a drug-induced AIH, there are some important clinical and immunological differences between CIP-induced hepatitis and genuine AIH. In most AIH cohorts, 80–90% of patients are females compared to 50–60% in CPI induced hepatotoxicity (European Association for the Study of the Liver, 2015; Shah et al., 2020). ANA/SMA is elevated in 80–90% in AIH but in very few patients with CPI-induced hepatotoxicity (European Association for the Study of the Liver, 2015; Shah et al., 2020).

Clinical guidelines from oncologists recommend high-dose corticosteroids in patients with moderate to severe CPI-induced hepatotoxicity. In grade II hepatotoxicity (ALT 3-5xULN), temporary interruption of CPI therapy is recommended and if liver tests return to baseline within 1–2 weeks, CPI can be resumed (Puzanov et al., 2017; Brahmer et al., 2018; Haanen et al., 2018). In patients with grade III or IV (>5x ULN) toxicity, corticosteroids should be given if there is no improvement after discontinuation of therapy and in case of a >10x ULN elevation permanent interruption is recommended. If the ALT elevation is accompanied by a rise in bilirubin, steroid treatment should be started immediately. Oral prednisone 0.5–1.0 mg/kg/day and starting iv methylprednisone 1–2 mg per day are recommended in grade 3–4 drug-induced hepatitis (Puzanov et al., 2017; Brahmer et al., 2018; Haanen et al., 2018).

These doses are much higher than those that are used in genuine AIH (European Association for the Study of the Liver, 2015). No clinical trial has been conducted to demonstrate the efficacy of steroids in general and high steroid doses in particular in this patient population. Guidline recommendations are mainly empirical and based on recommendations provided in the clinical trial protocols (Puzanov et al., 2017; Brahmer et al., 2018; Haanen et al., 2018).

De Martin et al. (2018) compared the pattern of DILI based on the CPI used, i.e. anti-programmed cell death protein 1(PD-1/PD ligand 1 (PD-L1) and anticytotoxic T lymphocyte antigen 4 (CTLA-4) monoclonal antibodies (Table 1). Overall, 10/16 patients (63%) were treated with corticosteroids, mainly receiving oral corticosteroids, whereas 6 (37%) improved spontaneously, and in three patients immunotherapy was reintroduced without recurrence of liver injury (De Martin et al., 2018). These results have mostly been reproduced by other groups (Gauci et al., 2018; Miller et al., 2020). In a large cohort from Texas, 33% of patients, and in a study from Paris, 50%, showed spontaneous improvement in liver tests (Gauci et al., 2018). In two studies, 14–17% of patients having been treated with steroids exhibited a recurrence of hepatotoxicity after tapering steroids (Pollack et al., 2018; Miller et al., 2020).

Based on the above mentioned studies, 50–67% of patients with CPI-associated DILI have been treated with steroids, and DILI rarely recurred after steroid tapering (De Martin et al., 2018; Gauci et al., 2018; Parlati et al., 2018; Miller et al., 2020).

In a study from Oxford, 19/21 patients (90.5%) with hepatotoxicity due to Check Point Inhibitors were treated with corticosteroids (Cheung et al., 2019) (Table 1), but only 50% had a treatment response, which was similar to a study from Japan (Kitagataya et al., 2020). The study by Ito et al. from Japan was somewhat of an outlier showing a response to steroid treatment in only 33% of cases (Ito t al. 2021) as shown in Table 1.

In a recent large retrospective cohort study from France consisting of 339 patients with advanced melanoma grade ≥3, hepatotoxicity was observed in 21 patients (Gauci et al., 2021). Thirteen patients were treated with steroids (steroid group, SG), whereas eight were not (non-steroid group, NSG). The median time for resolution of abnormal liver test was 49 days in the SG and 24 days in the NSG (p = NS). Only one patient had an unfavourable outcome. Two-year survival was 56% in the SG and 54% in the NSG (p = NS). Higher aminotransferases and bilirubin levels and lower prothrombin levels were observed in the SG than in the NSG (Gauci et al., 2021). The authors concluded that many patients with moderate to severe immune mediated hepatitis due to CPIs can be managed without steroids and suggested that steroid therapy should be considered in patients with high bilirubin and prothrombin values. A management protocol was proposed for validation in large, prospective cohorts (Gauci et al., 2021).

### Adverse Effects of Corticosteroid Treatment in Patients With DILI

There is little data published on adverse effects of high-dose corticosteroid treatment in patients with DILI. Generally, corticosteroid use can be associated with undesirable side effects, such as diabetes mellitus (DM), osteoporosis, hypertension, infections and psychosis. Most of these adverse effects are usually related to high doses and/or prolonged treatment and resolve after discontinuation of steroid treatment. Nevertheless, it seems that the administration of corticosteroids in DILI has not been reported as detrimental in these patients.

Mild adverse effects of corticosteroid treatment in patients with DILI have though been reported in some studies (Hu et al., 2016). Among 53 patients receiving corticosteroids for DILI, three patients developed respiratory infections (successfully treated with antibiotics), two patients with DM had uncontrolled increase of blood glucose, one experienced mental disturbances, and another one hypertension. Since steroids improved survival in that particular study and was associated with improvement in the liver injury, the authors strongly recommended short-term administration of steroids in severe DILI patients (Hu et al., 2016).

Safety of corticosteroid use for severe DILI was also investigated in a retrospective observational study enrolling 90 patients with severe DILI (Wan et al., 2019). Among them, 66 patients were receiving a median dose of 40 mg prednisone per day, while 24 patients composed the control group. The only adverse effects recorded were infections that occurred in 12 out of 66 (18%) patients, contrary to three out of 24 (12.5%) patients in the control group. However, Faje et al. (2018) reported that “high-dose” glucocorticoids (>7.5 mg prednisone or equivalent per day) for the treatment of ipilimumab-induced hypophysitis was associated with reduced survival in patients with melanoma. It was the first study reporting a potentially negative impact of corticosteroids on the efficacy of CPI treatment. In a large cohort study involving 740 melanoma patients receiving CPIs, Del Castillo et al. (2016) found that concomitant use of corticosteroids was associated with significantly higher risk of serious infections (odds ratio [OR], 7.71; 95% confidence interval [CI], 3.71–16.18; *p* < 0.0001).

In summary, corticosteroid administration in patients with DILI is usually well tolerated, although this has not been systematically reported. Some adverse effects of corticosteroids are worrisome, particularly if higher steroid doses are used, and clinicians should carefully evaluate each case for benefits and risks.

## Conclusion

Corticosteroids are often used in the treatment of DILI. However, their efficacy and safety are still disputable. Based on the available knowledge, patients with severe DILI or DI-AIH might benefit from steroid therapy, although evidence based on randomized controlled trials is largely lacking.

The global increase of CPI-based cancer treatment has led to a increase of indirect DILI. Hepatotoxicity occurs in 2–18% of patients treated with CPIs. Steroid therapy is not evidence based and its impact on patient outcomes is not clear. Therefore, indication, dose and duration of steroid therapy should be investigated in randomized controlled trials to fill the lack of evidence in the current treatment guidelines regarding efficacy, survival and side effects.

## Data Availability

The original contributions presented in the study are included in the article/Supplementary Material, further inquiries can be directed to the corresponding author.

## References

[B1] Bernal W. Ma Y. Smith H. M. Portmann B. Wendon J. Vergani D. (2007). The Significance of Autoantibodies and Immunoglobulins in Acute Liver Failure: a Cohort Study. J. Hepatol. 47 (5), 664–670. 10.1016/j.jhep.2007.05.011 17602781

[B2] Björnsson E. Jerlstad P. Bergqvist A. Olsson R. (2005). Fulminant Drug-Induced Hepatic Failure Leading to Death or Liver Transplantation in Sweden. Scand. J. Gastroenterol. 40 (9), 1095–1101. 10.1080/00365520510023846 16165719

[B3] Björnsson E. Talwalkar J. Treeprasertsuk S. Kamath P. S. Takahashi N. Sanderson S. (2010). Drug-Induced Autoimmune Hepatitis: Clinical Characteristics and Prognosis. Hepatology 51 (6), 2040–2048. 10.1002/hep.23588 20512992

[B4] Björnsson E. S. Gunnarsson B. I. Gröndal G. Jonasson J. G. Einarsdottir R. Ludviksson B. R. (2015). Risk of Drug-Induced Liver Injury from Tumor Necrosis Factor Antagonists. Clin. Gastroenterol. Hepatol. 13 (3), 602–608. 10.1016/j.cgh.2014.07.062 25131534

[B5] Björnsson E. S. Bergmann O. Jonasson J. G. Grondal G. Gudbjornsson B. Olafsson S. (2017). Drug-induced Autoimmune Hepatitis: Response to Corticosteroids and Lack of Relapse after Cessation of Steroids. Clin. Gastroenterol. Hepatol. 15 (10), 1635–1636. 10.1016/j.cgh.2017.05.027 28535988

[B6] Björnsson H. K. Gudbjörnsson B. Björnsson E. S. (2022). Infliximab-induced Liver Injury: Clinical Phenotypes, Autoimmunity and the Role of Corticosteroid Treatment. J. Hepatol. 76 (21), 86–92. Online ahead of print. 10.1016/j.jhep.2021.08.024 34487751

[B7] Borlak J. van Bömmel F. Berg T. (2018). N-acetylcysteine and Prednisolone Treatment Improved Serum Biochemistries in Suspected Flupirtine Cases of Severe Idiosyncratic Liver Injury. Liver Int. 38, 365–376. 10.1111/liv.13538 28782153

[B8] Brahmer J. R. Lacchetti C. Schneider B. J. Atkins M. B. Brassil K. J. Caterino J. M. (2018). Management of Immune-Related Adverse Events in Patients Treated with Immune Checkpoint Inhibitor Therapy: American Society of Clinical Oncology Clinical Practice Guideline. J. Clin. Oncol. 36 (17), 1714–1768. 10.1200/JCO.2017.77.6385 29442540 PMC6481621

[B9] Chalasani N. Fontana R. J. Bonkovsky H. L. Watkins P. B. Davern T. Serrano J. (2008). Causes, Clinical Features, and Outcomes from a Prospective Study of Drug-Induced Liver Injury in the United States. Gastroenterology 135, 1924–4. 10.1053/j.gastro.2008.09.011 18955056 PMC3654244

[B10] Cheung V. Gupta T. Payne M. Middleton M. R. Collier J. D. Simmons A. (2019). Immunotherapy-related Hepatitis: Real-World Experience from a Tertiary centre. Frontline Gastroenterol. 10 (4), 364–371. 10.1136/flgastro-2018-101146 31656561 PMC6788136

[B11] De Martin E. Michot J. M. Papouin B. Champiat S. Mateus C. Lambotte O. (2018). Characterization of Liver Injury Induced by Cancer Immunotherapy Using Immune Checkpoint Inhibitors. J. Hepatol. 68, 1181–1190. 10.1016/j.jhep.2018.01.033 29427729

[B12] Del Castillo M. Romero F. A. Argüello E. Kyi C. Postow M. A. Redelman-Sidi G. (2016). The Spectrum of Serious Infections Among Patients Receiving Immune Checkpoint Blockade for the Treatment of Melanoma. Clin. Infect. Dis. 63, 1490–1493. 10.1093/cid/ciw539 27501841 PMC5106605

[B13] European Association for the Study of the Liver (1979). Randomised Trial of Steroid Therapy in Acute Liver Failure. Report from the European Association for the Study of the Liver (EASL). Gut 20, 620–623. 10.1136/gut.20.7.620 385456 PMC1412526

[B14] European Association for the Study of the Liver (2015). EASL Clinical Practice Guidelines: Autoimmune Hepatitis. J. Hepatol. 63, 971–1004. 10.1016/j.jhep.2015.06.030 26341719

[B15] Faje A. T. Lawrence D. Flaherty K. Freedman C. Fadden R. Rubin K. (2018). High-Dose Glucocorticoids for the Treatment of Ipilimumab-Induced Hypophysitis Is Associated with Reduced Survival in Patients with Melanoma. Cancer 124 (18), 3706–3714. 10.1002/cncr.31629 29975414

[B16] Ferrero D. Pogliani E. M. Rege-Cambrin G. Fava C. Mattioli G. Dellacasa C. (2006). Corticosteroids Can Reverse Severe Imatinib-Induced Hepatotoxicity. Haematologica 91 (6 Suppl. l), ECR27. 10.3324/%x 16785130

[B17] Gauci M. L. Baroudjian B. Zeboulon C. Pages C. Poté N. Roux O. (2018). Immune-related Hepatitis with Immunotherapy: Are Corticosteroids Always Needed? J. Hepatol. 69, 548–550. 10.1016/j.jhep.2018.03.034 29747956

[B18] Gauci M. L. Baroudjian B. Bédérède U. Zeboulon C. Delyon J. Allayous C. (2021). Severe Immune-Related Hepatitis Induced by Immune Checkpoint Inhibitors: Clinical Features and Management Proposal. Clin. Res. Hepatol. Gastroenterol. 45 (2), 101491. 10.1016/j.clinre.2020.06.016 32773362

[B19] Germani G. Battistella S. Ulinici D. Zanetto A. Shalaby A. Pellone M. (2021). Drug Induced Liver Injury: from Pathogenesis to Liver Transplantation. Minerva Gastroenterol. 67 (1), 50–64. 10.23736/s2724-5985.20.02795-6 33222432

[B20] Ghabril M. Bonkovsky H. L. Kum C. Davern T. Hayashi P. H. Kleiner D. E. (2013). Liver Injury from Tumor Necrosis Factor-α Antagonists: Analysis of Thirty-Four Cases. Clin. Gastroenterol. Hepatol. 11, 558–e3. 10.1016/j.cgh.2012.12.025 23333219 PMC3865702

[B21] Haanen J. B. A. G. Carbonnel F. Robert C. Kerr K. M. Peters S. Larkin J. (2018). Management of Toxicities from Immunotherapy: ESMO Clinical Practice Guidelines for Diagnosis, Treatment and Follow-Up. Ann. Oncol. 29, iv264–iv266. 10.1093/annonc/mdy162 29917046

[B22] Hoofnagle J. H. Björnsson E. S. (2019). Drug-Induced Liver Injury - Types and Phenotypes. N. Engl. J. Med. 381, 264–273. 10.1056/NEJMra1816149 31314970

[B23] Hou F. Q. Zeng Z. Wang G. Q. (2012). Hospital Admissions for Drug-Induced Liver Injury: Clinical Features, Therapy, and Outcomes. Cell. Biochem. Biophys. 64, 77–83. 10.1007/s12013-012-9373-y 22806342

[B24] Hu P. F. Wang P. Q. Chen H. Hu X. F. Xie Q. P. Shi J. (2016). Beneficial Effect of Corticosteroids for Patients with Severe Drug-Induced Liver Injury. J. Dig. Dis. 17, 618–627. 10.1111/1751-2980.12383 27426618

[B25] Huffman B. M. Kottschade L. A. Kamath P. S. Markovic S. N. (2018). Hepatotoxicity after Immune Checkpoint Inhibitor Therapy in Melanoma: Natural Progression and Management. Am. J. Clin. Oncol. 41, 760–765. 10.1097/COC.0000000000000374 28749795

[B26] Ito T. Ishigami M. Yamamoto T. Mizuno K. Yamamoto K. Imai N. (2021). Clinical Course of Liver Injury Induced by Immune Checkpoint Inhibitors in Patients with Advanced Malignancies. Hepatol. Int. 15 (5), 1278–1287. 10.1007/s12072-021-10238-y 34373964

[B27] Karkhanis J. Verna E. C. Chang M. S. Stravitz R. T. Schilsky M. Lee W. M. (2014). Steroid Use in Acute Liver Failure. Hepatology 59 (2), 612–621. 10.1002/hep.26678 23929808 PMC4881740

[B28] Kirk A. P. Jain S. Pocock S. Thomas H. C. Sherlock S. (1980). Late Results of the Royal Free Hospital Prospective Controlled Trial of Prednisolone Therapy in Hepatitis B Surface Antigen Negative Chronic Active Hepatitis. Gut 21 (1), 78–83. 10.1136/gut.21.1.78 6988304 PMC1419564

[B29] Kitagataya T. Suda G. Nagashima K. Katsurada T. Yamamoto K. Kimura M. (2020). Prevalence, Clinical Course, and Predictive Factors of Immune Checkpoint Inhibitor Monotherapy-Associated Hepatitis in Japan. J. Gastroenterol. Hepatol. 35 (10), 1782–1788. 10.1111/jgh.15041 32187734

[B30] Miller E. D. Abu-Sbeih H. Styskel B. Nogueras Gonzalez G. M. Blechacz B. Naing A. (2020). Clinical Characteristics and Adverse Impact of Hepatotoxicity Due to Immune Checkpoint Inhibitors. Am. J. Gastroenterol. 115 (2), 251–261. 10.14309/ajg.0000000000000398 31789632

[B31] Ostapowicz G. Fontana R. J. Schiødt F. V. Larson A. Davern T. J. Han S. H. (2002). Results of a Prospective Study of Acute Liver Failure at 17 Tertiary Care Centers in the United States. Ann. Intern. Med. 137 (12), 947–954. 10.7326/0003-4819-137-12-200212170-00007 12484709

[B32] Pang L. Yang W. Hou F. (2018). Features and Outcomes from a Retrospective Study of 570 Hospitalized Chinese Patients with Drug-Induced Liver Injury. Clin. Res. Hepatol. Gastroenterol. 42, 48–56. 10.1016/j.clinre.2017.08.003 28927656

[B33] Parlati L. Vallet-Pichard A. Batista R. Hernvann A. Sogni P. Pol S. (2018). Incidence of Grade 3-4 Liver Injury under Immune Checkpoints Inhibitors: a Retrospective Study. J. Hepatol. 69 (6), 1396–1397. 10.1016/j.jhep.2018.08.014 30292476

[B34] Pollack M. H. Betof A. Dearden H. Rapazzo K. Valentine I. Brohl A. S. (2018). Safety of Resuming Anti-PD-1 in Patients with Immune-Related Adverse Events (irAEs) during Combined Anti-CTLA-4 and Anti-PD1 in Metastatic Melanoma. Ann. Oncol. 29, 250–255. 10.1093/annonc/mdx642 29045547 PMC5834131

[B35] Puzanov I. Diab A. Abdallah K. Bingham C. O. Brogdon C. Dadu R. (2017). Managing Toxicities Associated with Immune Checkpoint Inhibitors: Consensus Recommendations from the Society for Immunotherapy of Cancer (SITC) Toxicity Management Working Group. J. Immunother. Cancer 5 (1), 95–28. 10.1186/s40425-017-0300-z 29162153 PMC5697162

[B36] Rakela J. Mosley J. W. Edwards V. M. Govindarajan S. Alpert E. (1991). A Double-Blinded, Randomized Trial of Hydrocortisone in Acute Hepatic Failure. The Acute Hepatic Failure Study Group. Dig. Dis. Sci. 36 (9), 1223–1228. 10.1007/BF01307513 1716546

[B37] Reuben A. Koch D. G. Lee W. M. (2010). Drug-Induced Acute Liver Failure: Results of a U.S. Multicenter, Prospective Study. Hepatology 52 (6), 2065–2076. 10.1002/hep.23937 20949552 PMC3992250

[B38] Riveiro-Barciela M. Barreira-Díaz A. Vidal-González J. Muñoz-Couselo E. Martínez-Valle F. Viladomiu L. (2020). Immune-related Hepatitis Related to Checkpoint Inhibitors: Clinical and Prognostic Factors. Liver Int. 40, 1906–1916. 10.1111/liv.14489 32329119

[B39] Rodrigues S. Lopes S. Magro F. Cardoso H. Horta e Vale A. M. Marques M. (2015). Autoimmune Hepatitis and Anti-tumor Necrosis Factor Alpha Therapy: A Single center Report of 8 Cases. World J. Gastroenterol. 21, 7584–7588. 10.3748/wjg.v21.i24.7584 26140007 PMC4481456

[B40] Shah P. Sundaram V. Björnsson E. (2020). Biologic and Checkpoint Inhibitor-Induced Liver Injury: A Systematic Literature Review. Hepatol. Commun. 4, 172–184. 10.1002/hep4.1465 32025603 PMC6996412

[B41] Stravitz R. T. Lefkowitch J. H. Fontana R. J. Gershwin M. E. Leung P. S. Sterling R. K. (2011). Autoimmune Acute Liver Failure: Proposed Clinical and Histological Criteria. Hepatology 53 (2), 517–526. 10.1002/hep.24080 21274872 PMC3080034

[B42] Suzuki A. Brunt E. M. Kleiner D. E. Miquel R. Smyrk T. C. Andrade R. J. (2011). The Use of Liver Biopsy Evaluation in Discrimination of Idiopathic Autoimmune Hepatitis versus Drug-Induced Liver Injury. Hepatology 54 (3), 931–939. 10.1002/hep.24481 21674554 PMC3192933

[B43] Wan Y. M. Wu J. F. Li Y. H. Wu H. M. Wu X. N. Xu Y. (2019). Prednisone Is Not Beneficial for the Treatment of Severe Drug-Induced Liver Injury: an Observational Study (STROBE Compliant). Medicine (Baltimore) 98, e15886. 10.1097/MD.0000000000015886 31261497 PMC6616446

[B44] Ware A. J. Jones R. E. Shorey J. W. Combes B. (1974). A Controlled Trial of Steroid Therapy in Massive Hepatic Necrosis. Am. J. Gastroenterol. 62, 130–133. 4606110

[B45] Weber S. Benesic A. Rotter I. Gerbes A. L. (2019). Early ALT Response to Corticosteroid Treatment Distinguishes Idiosyncratic Drug-Induced Liver Injury from Autoimmune Hepatitis. Liver Int. 39, 1906–1917. 10.1111/liv.14195 31319011

[B46] Wei G. Bergquist A. Broomé U. Lindgren S. Wallerstedt S. Almer S. (2007). Acute Liver Failure in Sweden: Etiology and Outcome. J. Intern. Med. 262 (3), 393–401. 10.1111/j.1365-2796.2007.01818.x 17697161

[B47] Wree A. Dechêne K. Herzer W. K. Hilgard A. Syn W. K. Gerken G. (2011). Steroid and Ursodesoxycholic Acid Combination Therapy in Severe Drug-Induced Liver Injury. Digestion 84, 54–59. 10.1159/000322298 21304237

